# *Streptococcus pneumoniae* in Saliva of Dutch Primary School Children

**DOI:** 10.1371/journal.pone.0102045

**Published:** 2014-07-11

**Authors:** Anne L. Wyllie, Mei Ling J. N. Chu, Mariëlle H. B. Schellens, Jody van Engelsdorp Gastelaars, Marc D. Jansen, Arie van der Ende, Debby Bogaert, Elisabeth A. M. Sanders, Krzysztof Trzciński

**Affiliations:** 1 Department of Paediatric Immunology and Infectious Diseases, Wilhelmina's Children Hospital, University Medical Centre Utrecht, Utrecht, The Netherlands; 2 Brain Center Rudolf Magnus, Department of Neurology, University Medical Center Utrecht, Utrecht, The Netherlands; 3 Department of Medical Microbiology and the Netherlands Reference Laboratory for Bacterial Meningitis, Academic Medical Center, Amsterdam, The Netherlands; Rockefeller University, United States of America

## Abstract

While nasopharyngeal sampling is the gold standard for the detection of *Streptococcus pneumoniae* carriage, historically seen, saliva sampling also seems highly sensitive for pneumococcal detection. We investigated *S. pneumoniae* carriage in saliva from fifty schoolchildren by conventional and molecular methods. Saliva was first culture-enriched for pneumococci, after which, DNA was extracted from all bacterial growth and tested by quantitative-PCR (qPCR) for pneumococcus-specific genes *lytA* and *piaA*. Next, serotype composition of the samples was determined by serotype-specific qPCRs, conventional-PCRs (cPCR) and sequencing of cPCR amplicons. Although only 2 (4%) of 50 samples were positive by conventional diagnostic culture, 44 (88%) were positive for pneumococci by qPCR. In total, we detected the presence of at least 81 pneumococcal strains representing 20 serotypes in samples from 44 carriers with 23 carriers (52%) positive for multiple (up to 6) serotypes. The number of serotypes detected per sample correlated with pneumococcal abundance. This study shows that saliva could be used as a tool for future pneumococcal surveillance studies. Furthermore, high rates of pneumococcal carriage and co-carriage of multiple pneumococcal strains together with a large number of serotypes in circulation suggests a ubiquitous presence of *S. pneumoniae* in saliva of school-aged children. Our results also suggest that factors promoting pneumococcal carriage within individual hosts may weaken competitive interactions between *S. pneumoniae* strains.

## Introduction

*Streptococcus pneumoniae* is a commensal bacterium of the upper respiratory tract, with asymptomatic colonisation of the nasopharynx considered to be a pre-requisite to respiratory and invasive pneumococcal disease [1], [2]. Pneumococcal disease is targeted by conjugated polysaccharide vaccines (PCV) which induce specific anti-capsular antibodies against the vaccine serotypes (VTs). However, the net benefit of vaccination is reduced by an increase in disease caused by non-vaccine serotypes (NVTs) [3], [4]. Since vaccine-induced shifts in disease are reflected by a redistribution of serotypes in carriage, epidemiological surveillance on asymptomatic colonisation is widely used to monitor the effects of vaccination and the emergence of NVTs [5].

The current standard procedure in epidemiological studies on pneumococcal carriage is the conventional culture of a nasopharyngeal swab and isolation of live *S. pneumoniae* followed by serotype determination with type-specific sera [5]. This method however, has been shown to have limitations for the detection of carriage of multiple pneumococcal strains in individuals, with co-colonisation rates thought to be largely underestimated [6]. Identification of all strains carried by a host, including those in lesser abundance, should be an important consideration for surveillance studies, as this may provide insight into the dynamics of pneumococcal colonisation and help to explain the mechanisms behind vaccine-induced redistribution of serotypes seen in carriage and replacement of VTs with NVTs in pneumococcal disease [7]. Epidemiological studies in humans [8], [9] and experimental studies in animals [10]–[12] point towards competitive interactions between serotypes as major drivers in those dynamics.

Saliva has a record as being one of the most sensitive sampling methods for pneumococcal carriage detection [13]. The polymicrobial nature of saliva however, makes detection of *S. pneumoniae* by conventional culture nearly impossible. This limitation was addressed in early studies by inoculating animals susceptible to pneumococcal infection with saliva, followed by isolating *S. pneumoniae* from the blood of the sick animals [14]–[16]. Contemporary serological and molecular diagnostic methods now allow for accurate detection of pneumococcal carriage [17], [18] and for identification of serotypes present, independently from sample type, including those from polymicrobial settings [9], [19]–[21].

Here, we investigated saliva carriage in a cross-sectional study conducted in fifty Dutch schoolchildren. In order to address the poor sensitivity of conventional diagnostic cultures when applied to detect *S. pneumoniae* in saliva, we used PCR-based methods of carriage and serotype detection and tested samples enriched for pneumococcal presence. Saliva carriage was common with almost 90% of children positive for *S. pneumoniae* and a co-carriage rate of 52%, with the number of serotypes detected per sample correlating with the density of *S. pneumoniae* in saliva.

## Materials and Methods

### Ethics statement

Parents of the schoolchildren were informed in writing about the purpose and procedure of the study, and were asked to give verbal informed consent for their child to participate. Parental consent was collected in the presence of two independent witnesses. Parents who objected were to be registered in writing, but all the parents consented to their child's participation in the study. Except for the child's age and what class they attended no information was recorded. Since saliva collection is non-invasive, does not cause discomfort and due to the fact that no information permitting child identification was recorded, the medical ethics committee of the Wilhelmina's Children Hospital, University Medical Centre Utrecht decided the study did not fall within the ambit of the WMO (Medical Research involving Human Subject Act, www.fmwv.nl). Consequently, the ethics committee approved the consent procedure and granted an official waiver for further ethical review.

### Study settings

The study was conducted on a single school day in June 2012 at a rural school of 190 students, in the province of Utrecht. Fifty students attending two classes took part in the study. The older class consisted of 24 students aged 8 to 10 years (median 9) and the younger class consisted of 26 students, 5 to 8 years old (median 7).

### Sample collection

Students spat into a 15 ml falcon tube (BD, The Netherlands). Samples were placed on wet ice and within 4 hours transported to the lab. On arrival, samples were vigorously vortexed for 20 seconds and 10 µl cultured on trypticase soy agar supplemented with 7% defibrinated sheep blood and gentamicin 5 mg/l (SB7-Gent, Oxoid, Badhoevedorp, The Netherlands). The remaining raw saliva was stored frozen at −80°C in 10% glycerol. After overnight incubation at 37°C and 5% CO_2_, the SB7-Gent plates were inspected and colonies suspected to be *S. pneumoniae* were isolated and tested for susceptibility to optochin and bile solubility [5]. All remaining bacterial growth was harvested into 10% glycerol in Brain Heart Infusion (Oxoid, Bedhoverdorp, the Netherlands) and stored frozen at −80°C as previously described [18]. We considered the harvested samples as culture-enriched for *S. pneumoniae*.

### Isolation of bacterial DNA

DNA was extracted from 100 µl of raw saliva and 200 µl of culture-enriched samples using a modified mag forensics extraction kit protocol (LGC Genomics, Berlin, Germany). All samples were thawed on ice, vigorously vortexed for 20 seconds then transferred to 1.5 ml Eppendorf tubes containing 650 µl 0.1 mm zirconium beads (Biospec Products, Bartlesville, OK) in lysis buffer and 550 µl phenol (Sigma-Aldrich, St Louis, MO). Samples were mechanically disrupted through two cycles of 2 minutes bead beating (Mini-Beadbeater-24, Biospec Products) and 2 minutes on ice, before centrifugation at 12,000×g for 2 minutes. The aqueous DNA phase was then transferred to new Eppendorf tubes pre-filled with 10 µl of magnetic particle suspension in 1.3 ml binding buffer. Following 30 minutes of incubation in a shaker at room temperature, the beads were washed with 200 µl wash buffers 1 and 2, and then dried for 20 minutes at 55°C. DNA was eluted in 50 µl template volumes and stored at +4°C. The procedure was repeated when more template was required.

### Molecular quantification of bacteria

Quantification of total bacterial DNA load in raw saliva samples was determined by 16S based real-time quantitative PCR (qPCR) as previously described [22].

### Molecular detection of *S. pneumonia*

All DNA templates were tested by qPCRs specific for *S. pneumoniae* genes *lytA* [17] and *piaA* [18] *and* were considered positive when both genes were <40 C*_T_*. In all qPCRs, 2.5 µl DNA template was tested in 25 µl reaction volumes.

### Sample serotype determination with molecular methods

First, serotype-specific sequences were detected with qPCR in DNA extracted from culture-enriched samples using primers and probes as published by Azzari *et al*. [21], targeting 21 serotypes or serogroups. Samples were considered positive when any signal (<45 C*_T_*) was present. Next, serotype-specific sequences were detected by sequential multiplex PCR (smPCR) using Qiagen Multiplex PCR kit (QIAgen, Venlo, The Netherlands) and 39 primer pairs published by Pai *et al*. [23] and Carvalho *et al*. [20], targeting individual serotypes or serotype clusters (69 serotypes in total). Details of the method are available online (http://www.cdc.gov/ncidod/biotech/strep/pcr.htm). DNA of clinical pneumococcal strains of the serotypes tested in the assay were included as positive controls in smPCR at a concentration of 1 ng/µl and five 10-fold dilutions (from 10^0^ to 10^−4^) in every qPCR run, in order to generate a standard curve.

All samples positive for serotype-specific product in smPCR were tested in conventional PCR (cPCR) with a single pair of serotype-specific primers, after which, amplicons were sequenced to confirm the serotype. Samples were considered positive for the cPCR serotype when the sequenced product had full homology (no single nucleotide mismatch detected) with either the published *S. pneumoniae* sequence or the sequence of the control pneumococcal strain.

### Recovery of *S. pneumoniae* from culture harvests

Isolation of *S. pneumoniae* from culture-enriched samples was performed as previously described [18]. In short, culture-enriched saliva samples generating *S. pneumoniae*-specific signal by qPCR were thawed and 100 µl of 10^−3^ and 10^−4^ dilutions were cultured on tryptic soy agar supplemented with 5% defibrinated sheep blood in a second attempt to recover live pneumococci, selecting putative pneumococcal colonies to be tested for *S. pneumoniae* as described above. If present, a minimum 8 colonies per sample were re-cultured and tested.

### Quellung reaction

*S. pneumoniae* strains were serotyped at the Regional Laboratory of Public Health in Haarlem using the Quellung method and serotype specific sera (StatenserumInstitut, Copenhagen, Denmark).

### Statistical methods

Statistical analyses were conducted using GraphPad Prism V5.0 (GraphPad Software, San Diego, CA, USA). Statistical significance was defined as a p value ≤0.05.

## Results

### Isolation of live pneumococci

Live *S. pneumoniae* strains were isolated from 2 out of 50 (4%) samples cultured on the day of saliva collection. Extensive re-culturing of culture-enriched samples lead to the isolation of *S. pneumoniae* from an additional 9 saliva samples and increased the overall number of schoolchildren positive by culture for *S. pneumoniae* to 11 out of 50 (22%). One child was culture-positive for two morphologically different pneumococcal strains. All 12 isolates were stored for further analysis.

### Detection of *S. pneumoniae* with molecular methods

**Figure 1:**
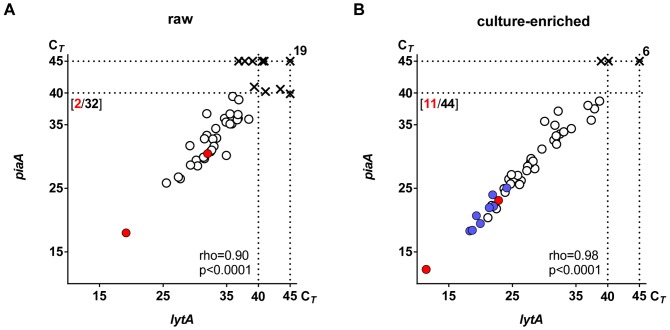
Detection of *Streptococcus pneumoniae* in saliva samples from schoolchildren. Results of qPCR-based detection of *S. pneumoniae*-specific genes *lytA* and *piaA* and of live *S. pneumoniae* isolation from raw (**A**) and culture-enriched (**B**) samples of saliva collected from 50 schoolchildren. Each dot or cross represents an individual sample. The position of symbols corresponds to C*_T_* values for *lytA*- and *piaA*-specific signals as marked on corresponding axes. Dots represent samples classified as positive for *S. pneumoniae* with qPCR. Red dots represent samples from which live pneumococci were isolated from primary cultures of raw saliva on gentamicin-supplemented plates inoculated on the day of sample collection (**A** and **B**). Blue dots represent additional samples from which live pneumococci were isolated from re-cultured gentamicin plate culture harvests (**B**). Crosses represent samples classified by qPCR as negative for *S. pneumoniae*. Dotted lines mark the threshold assigned to discriminate between positive (C*_T_*<40) and negative samples, and the total number of 45 cycles in each qPCR reaction. Numbers in square brackets depict (in red) the number cultures from which live *S. pneumoniae* was isolated and (in black) number of samples with C*_T_* values <40 for both targeted genes. The number in the upper right corner depicts the number of samples classified with qPCR as negative for *S. pneumoniae*. Spearman's rank correlation coefficient (rho) and the associated *P* value (p) for samples generating any *lytA*- or *piaA*-specific signal (C*_T_*<45) are shown.

**Figure 2:**
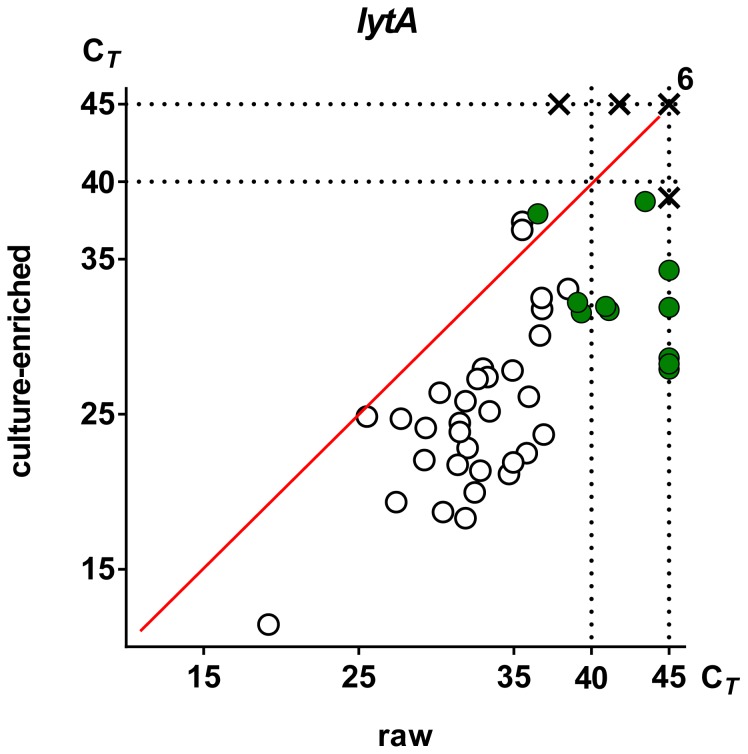
Impact of culture-enrichment on qPCR detection of *Streptococcus pneumoniae* gene *lytA* in saliva from schoolchildren. Each dot or cross represents an individual sample. The position of symbols corresponds to C*_T_* values for *lytA*-specific signals in DNA extracted from raw and culture-enriched sample of saliva as marked on corresponding axes. Dots represent 44 samples classified as positive and crosses represent 6 samples classified as negative for *S. pneumoniae* with qPCR. Open dots represent 32 saliva samples classified as positive *for S. pneumoniae* in both raw and culture-enriched samples. Green dots represent 12 samples classified as positive only after culture-enrichment. Dotted lines mark the threshold assigned to discriminate between positive (C*_T_* <40) and negative samples, and the total number of 45 cycles in each qPCR reaction. There was a significant increase in quantity of both *lytA* and (not shown) *piaA* detected with qPCR in culture-enriched compared to raw saliva samples (Mann-Whitney, p = <0.0001).

Thirty-two of 50 (64%) saliva samples were identified as positive for *S. pneumoniae* when DNA extracted from raw saliva was tested by qPCR (Figure 1A). This number increased to 44 of 50 (88%) samples when DNA extracted from culture-enriched samples was processed by qPCR (Figure 1B). Significantly fewer students were identified as *S. pneumoniae* carriers by qPCR in the older class (18 of 24, 75%) compared to all 26 (100%) students of the younger class (Fisher Exact, p<0.01). All samples positive for *S. pneumoniae* in raw saliva were also positive in culture-enriched samples and there was a significant increase in quantity of *S. pneumoniae*-specific sequences detected with qPCR in culture-enriched compared to raw saliva samples (Mann-Whitney, p<0.0001; Figure 2). Furthermore, we also tested if *lytA* could be detected in saliva samples collected from non-carriers, sham-inoculated with pneumococcal DNA before plating. Only traces of *lytA* were detected in harvests of samples spiked with high quantities of *S. pneumoniae* DNA (Figure S1). This and the increase in *S. pneumoniae*-specific signal in DNA extracted from culture-enriched compared to raw saliva point at viable bacteria as the source of pneumococcal sequences detected with qPCR.

### Serotype carriage in saliva detected with molecular methods

**Figure 3:**
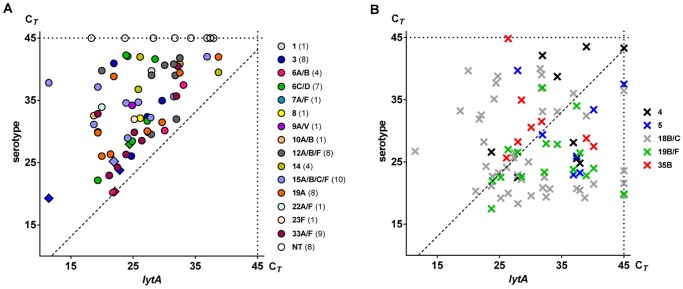
Presence of serotype-specific *Streptococcus pneumoniae* DNA sequences detected with qPCR in saliva samples from schoolchildren. Results of capsular gene detection in DNA extracted from culture-enriched samples of saliva collected from 50 children in assays considered as reliable (**A**) in the study or as unreliable (**B**) due to lack of specificity as determined by being no more than 2 C*_T_* stronger than the matching signal for the pneumococcal gene *lytA* in the same sample (dashed line). Each symbol represents individual serotype-specific signal detected with qPCR. Position of the symbol corresponds to the C*_T_* value for *lytA-*specific signal considered to be representative of overall quantity of *S. pneumoniae* in a sample (X-axis) and to the C*_T_* value for a particular serotype-specific signal (Y-axis). Results are colour-coded per serotype as shown in the legend. In **A**, diamonds represent samples from which a strain of a particular serotype was cultured, coloured dots represent samples culture-negative but qPCR positive for a serotype and open dots depict samples from carriers but negative in qPCR for any serotype-specific signal thus considered qPCR-non-typeable (NT). Numbers in brackets next to a serotype symbol in the legend report the total number of samples classified as positive in serotype-specific qPCR. In **B**, crosses depict serotype-specific signals detected in assays classified as unreliable in the study. Note that in all five qPCR assays there were a number of samples with a signal specific for serotype considerably stronger than for *lytA*.

DNA extracted from culture-enriched samples was tested for the presence of serotype-specific sequences using two molecular methods, qPCR and smPCR. All 50 samples were analysed regardless of whether *S. pneumoniae* was detected by *lytA* and *piaA* qPCRs. We expected that the signal for a serotype would not be stronger than the signal for *S. pneumoniae* in qPCR, however due to minor inter-assay variations in sensitivity, we considered the results of serotype-specific qPCR assays as reliable if the serotype-specific signal was no more than 2 C*_T_* stronger than the matching signal for the pneumococcal gene *lytA* (Figure 3A). All results of any assay giving serotype-specific signal >2 C*_T_* stronger than the matching *lytA* signal of a sample, were excluded from analysis due to the lack of assay specificity. This resulted in the exclusion of results from qPCR assays targeting serotypes 4, 5, 18B/18C, 19B/19F and 35B (Figure 3B). In the remaining qPCR assays we detected 65 positive signals representing 15 *S. pneumoniae* serotypes/serogroups in 36 out of 44 saliva samples from carriers (Figure 3A).

Subsequently, smPCR followed by cPCR product sequence analysis, lead to the identification of 48 serotype-specific amplicons representing 15 serotypes/serogroups in 27 out of 44 carriers. Conversely, individual assays that resulted in amplicons without full homology (one or more nucleotide mismatches) to either the published or control strain sequences were considered falsely positive and excluded (Table 1).

**Table 1:** Summary of the specificity of molecular methods used in the study to determine the sample serotype composition.

Method	Serotypes targeted in the assays	Result and interpretation
qPCR	1, 3, 6A/B/C/D, 7A/F, 8, 9A/V, 10A/B, 12A/B/F, 14, 15A/B/C/F, 19A, 22A/F, 23F, 33A/F	all results reliable* ^A^
	4, 5, 18B/C, 19B/F, 35B	all results excluded^B^
	20, 38	not detected
sequencing of cPCR product	6A/B/C/D, 9A/V, 16F, 19A, 23B, 23F	all results reliable^C^
	3, 7F/A, 10A, 11A/D, 15A/F, 15B/C, 21, 33F/A/37, 35F/47F	some results reliable^D^
	2, 4, 5, 7C/B/40, 10F/C/33C, 12F/A/B/44/46, 13, 17F, 18C/F/B/A, 20, 22F/A, 23A, 35B, 39	all results excluded^E^
	1, 8, 9N/L, 14, 19F, 24F/A/B, 31, 34, 35A/C/42, 38/25F/A	not detected

* – results of the molecular assays targeting this set of serotypes were used in the analysis of method sensitivity in the study

A – serotype-specific signal no more than 2 C*_T_* stronger for the matching *lytA* signal from the sample.

B – insufficient assay specificity.

C – sequence of full homology (no single nucleotide mismatch) with either the sequence published for *S. pneumoniae* or the sequence detected in *S. pneumoniae* control strain tested in the study.

D – sequence included when fully homologous with either the sequence published for *S. pneumoniae* or with the sequence detected in *S. pneumoniae* control strain tested in the study and excluded when not fully homologous.

E - absence of full homology with *S. pneumoniae* sequences.

Serotype-specific assays considered as reliable in both the qPCR and the smPCR/sequencing hybrid method (Table 1, serotype-specific assays listed in the top row only) were used to compare the sensitivity of molecular methods of serotype detection. Altogether we detected 67 serotypes when samples were tested with this particular subset of assays. Sixty-five (97%) of 66 were detected using qPCR and 38 (57%) using the hybrid method, implying 40% lower sensitivity of smPCR compared to qPCR (p<0.001).

Altogether, after exclusion of results considered unreliable, we detected 113 serotype-specific signals in 41 of 44 (93%) samples from carriers (Table S1). The remaining three samples were considered PCR non-typeable (NT). In 35 cases a positive qPCR result was matched by a positive result by smPCR. On top of this, 30 signals were detected only by qPCR and 13 only by smPCR. Eleven of these 13 were detected in smPCR assays with no corresponding serotype-specific qPCR assay used in the study.

**Figure 4:**
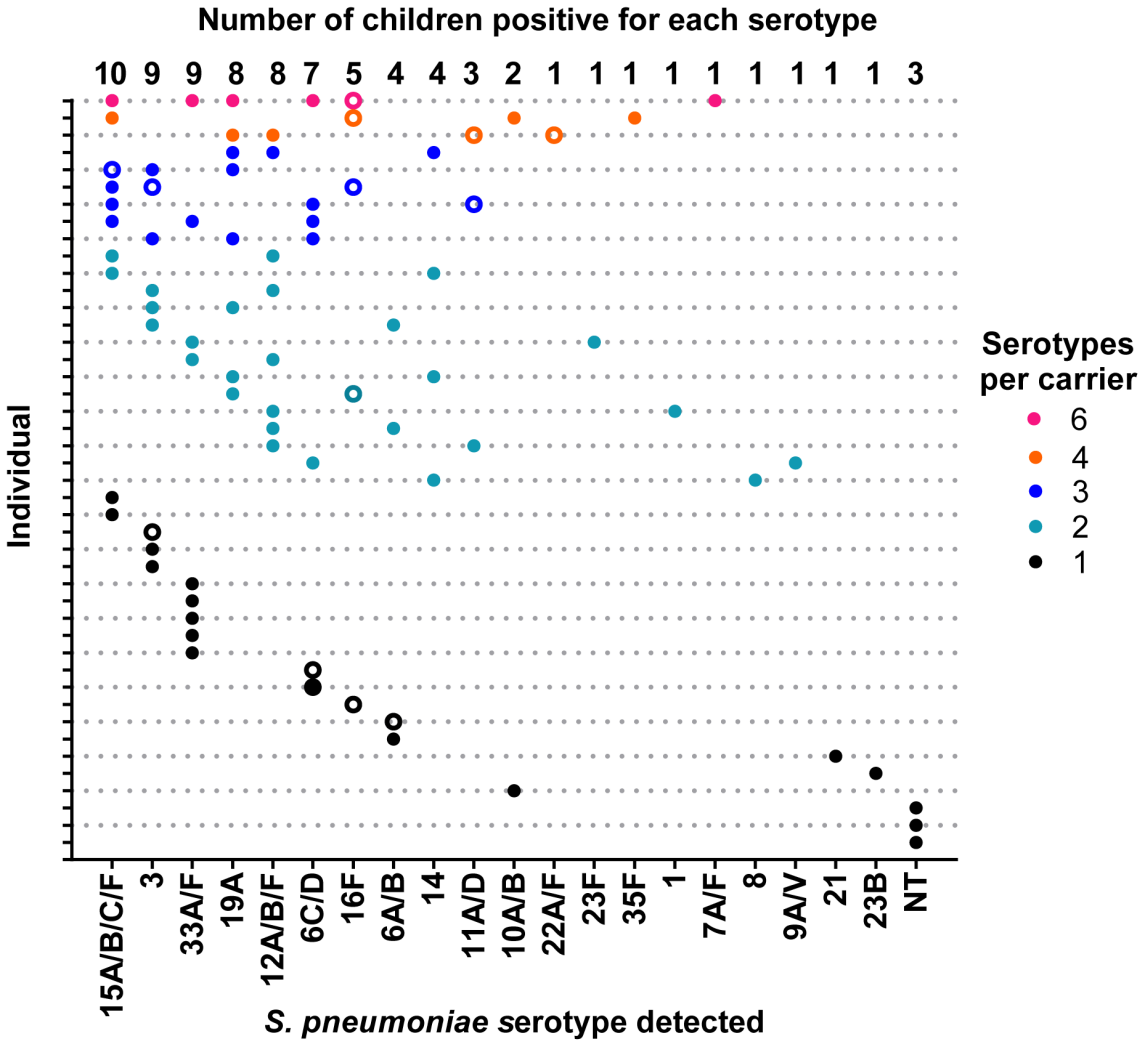
*Streptococcus pneumoniae* serotype carriage in saliva of schoolchildren. Serotypes detected are ranked by decreasing number of samples classified as positive in the study (upper X-axis). Every y-axis interval tick represents an individual carrier. Carriers are ranked according to decreasing number of serotypes detected as stated in the figure legend. Open circles depict strains of *S. pneumoniae* cultured from a sample (serotypes 3, 6A, 6C, 15B, 11A and 16F). NT – samples classified as positive for *S. pneumoniae* but as negative for the presence of any of the serotypes tested for.

We interpreted the results as evidence of a genuine presence of sequences specific for a minimum of 81 carried *S. pneumoniae* strains including 78 strains representing one of 20 serotypes and 3 NT strains (Figure 4). Twenty-three of the 44 (52%) samples from carriers were positive for more than one serotype/serogroups (range from 2 to 6). Overall, the mean number of strains per carrier was 1.84 (median 2). There was a significant correlation between the number of serotypes detected and strength of *S. pneumoniae-*specific signal in raw saliva (Spearman's correlation test for non-parametric data, rho = 0.54, n = 49, *p*<0.0001 for both *lytA* and *piaA*), but as expected, not with the total bacterial load as quantified by 16S specific qPCR (rho = 0.21, n = 49, *p* = 0.15) (Figure S2). The results of serotype detection by molecular methods were in full concordance with the Quellung results for all 12 cultured strains.

None of 12 cultured *S. pneumoniae* isolates was of a serotype targeted by the 10-valent pneumococcal conjugated vaccine (PCV10; serotypes 1, 4, 5, 6B, 7F, 9V, 14, 18C, 19F and 23F) used in the Dutch National Immunisation Programme (NIP) at the time of the study. Since we considered PCR assays targeting VTs 4 and 5 as unreliable (Table 1), we were only able to detect the presence of 8 out of 10 PCV10 serotypes by using molecular methods. Six (8%) of 80 strains detected in total belonged to one of the 8 targeted PCV10 serotypes; 1 (n = 1), 14 (n = 4) and 23F (n = 1). Another five represented 6A/6B (n = 3), 7A/7F (n = 1) and 9A/9V (n = 1) though an exact distinction between VTs (6B, 7F and 9V) and NVTs could not be made.

## Discussion

We applied molecular methods to study pneumococcal carriage in saliva collected from 50 schoolchildren attending a rural school near Utrecht. We found that the majority of children tested positive for *S. pneumoniae* presence (44 of 50, 88%), with the majority of carriers (23 of 44, 52%) positive for the presence of more than one of the 20 serotypes of *S. pneumoniae* identified in this study, despite our stringent protocol for exclusion of potential false positive results.

Oral fluids are arguably the most polymicrobial of all samples used in surveillance on pneumococcal carriage. Furthermore, the oral cavity is the ecological niche for many other streptococcal species phenotypically similar to *S. pneumoniae* [24]. This affects both the sensitivity and specificity of both culture and molecular methods in the detection of pneumococcal carriage. Reports from epidemiological studies employing molecular tests along with conventional diagnostic culture methods for pneumococcal carriage detection uniformly report on the higher sensitivity of molecular tests [18], [19], [21], [25]. However, the specificity of culture-independent methods is questioned since homologues of genes targeted in molecular assays are known to also be present in other streptococcal species [24], [26]–[28]. In order to increase the specificity of carriage detection in our study, all samples were simultaneously tested for the presence of two conserved pneumococcal genes *lytA* and *piaA*. To our knowledge non-pneumococcal strains have never tested positive in either of the *lytA* or *piaA* assays despite *lytA* now being widely used [18], [20], [24], [29]. Furthermore, the high correlation between results of *lytA* and *piaA* quantification observed by us further supports the specificity of both tests.

Our *S. pneumoniae* carriage rate of 88% is one of the highest reported for any population and largely surpasses results from contemporary culture-based surveillances on nasopharyngeal carriage in school-age children [1], [30]. It is however in line with historical data from studies detecting *S. pneumoniae* in saliva or in throat washes using a sensitive mouse inoculation method [14]. For example, in a longitudinal study conducted in Germany in 1932, Gundel and Okura [31] reported 66% (471 of 715) saliva samples from teenagers attending sex-segregated schools to be positive for *S. pneumoniae* with point prevalence up to 85% amongst boys and up to 71% amongst girls. Also, Rosenau *et al*. [32] reported a uniform 50% rate of oropharyngeal carriage in Boston, MA, amongst normal (asymptomatic) individuals independent of subjects age [32]. Since *S. pneumoniae* strains vary greatly in their virulence in mice [33]–[35] pneumococcal carriage is likely to be underestimated in these historic studies whereas the strains virulence would have limited effect on the current sensitivity of molecular methods.

Similar to recently published papers by Carvalho *et al*. [24], [29] our study also highlights the limitation of the current molecular methods designed for the detection of serotypes when applied to samples from the oral niche. The number of non-specific signals generated when using molecular methods in both this study and as reported by Carvalho *et al*. [24], [29], exemplifies the need for further optimisation of culture-independent methods for serotype-detection and underlines the importance of analysing samples from non-carriers in order to identify tests of poor specificity. For smPCR, the accuracy of the assay will raise proportionally to the number of samples from non-carriers tested. The quantitative nature of qPCRs may allow reaching similar levels of confidence by testing fewer samples and comparing quantities of species- versus serotype-specific sequences. This, as well as the higher sensitivity of qPCR supports the supremacy of this method over cPCR.

We detected serotype-specific signals in 41 of the 44 (93%) carriers with over half of the carriers being positive for two or more serotypes. The co-colonisation rate may be still underestimated in our study as only 21 of all pneumococcal serotypes were tested for by the more sensitive qPCR method [21]. Also, by exclusion of all results from PCRs that were considered unreliable, we may have dismissed genuine results. Furthermore, strong false-positive signal could mask a weaker signal from *S. pneumoniae* strains if present, and finally the method was not suited to detect un-encapsulated pneumococcal strains. Despite this, a 52% rate of multistrain carriage is amongst the highest ever reported. Interestingly, an exceptionally high 35% rate of saliva co-carriage was also described by Gundel and Okura in 1932, reporting point rates of co-colonisation of up to 62% among boys and 55% among girls despite their study not being designed to detect more than three co-carried pneumococcal strains [31].

Since we relied on culture-independent methods and no live *S. pneumoniae* was isolated from the majority of samples that were classified as positive, we might also have overestimated the number of strains present in saliva [24]. In line with this, false serotype-specific signals from confounding, non-pneumococcal bacteria would be expected to be discordant with species-specific signal. Furthermore, a higher number of confounding signals would be expected in samples intrinsically richer in bacteria. We found on the contrary that there was no correlation between the number of strains present and the total bacterial load of raw saliva but we observed a correlation between the number of strains detected and the quantity of *S. pneumoniae* by *lytA* and *piaA* in the raw saliva samples.

Relatively few of the saliva samples tested in the study were positive for serotypes targeted by the PCV10 vaccine. Although none of the children were immunised with PCV10 and only part with the 7-valent PCV vaccine (PCV7), the low prevalence of VTs is most probably a consequence of herd effects since PCV7 and PCV10 were introduced for newborns in the Dutch NIP in 2006 and 2011, respectively (35). Interestingly, up to a quarter of pneumococcal strains detected (21 of 81, or 26%) were of serotypes 3, 6A or 19A targeted by the thirteen-valent vaccine (PCV13) only, which is not used in the Dutch NIP.

High rates of carriage and co-colonisation, together with a relatively high number of 20 serotypes in circulation reflects a ubiquitous presence *of S. pneumoniae* in this group of children. Interestingly, a recent genome wide transposon mutagenesis study revealed that *S. pneumoniae* is well adapted to growth in human saliva and identified genes crucial for this adaptation [36]. The same study showed that pneumococcus survives well in saliva at various temperatures with and without CO_2_ supporting a role of saliva in pneumococci transmission between carriers. These findings highlight the need for more studies on the *S. pneumoniae* reservoir and pathogenesis in human saliva.

Since *S. pneumoniae* is relatively resistant to desiccation [37], the high carriage rates we observed in this study could reflect environmental exposure and presence of non-viable bacteria or pneumococcal DNA rather than genuine carriage events. Although we did not culture *S. pneumoniae* from the majority of samples classified as positive by molecular methods, higher quantities of *S. pneumoniae-*specific genes detected in culture-enriched compared to raw saliva samples and the disappearance of extra-cellular *lytA* from samples sham-inoculated with pneumococcal DNA support presence of live pneumococci in saliva from carriers.

If the results of our study accurately reflect the typical circulation of pneumococcal strains carried by schoolchildren, this high rate of co-carriage and high diversity of serotypes suggest rather weak, competitive interactions between pneumococcal strains at the level of individual carriers. Interestingly, in a longitudinal epidemiological study on *S. pneumoniae* carriage in Danish children, Mehtälä *et al*. [8] observed decelerated clearance of pneumococcal carriage in co-colonised children compared to individuals carrying a single strain. Also, a decelerated rate of carriage clearance was recently observed by Marks *et al*. [38], in a mouse model of pneumococcal co-colonisation. This suggests the presence of inter-strain collaboration or host-specific factors predisposing for carriage, hence multistrain colonisation and decelerated carriage clearance. It also implies that certain factors may universally promote *S. pneumoniae* carriage in some individuals, resulting in species-dominated profiles in the microbiota of the upper airways of asymptomatic children [39].

The limitation of this study is its cross-sectional nature. With samples collected from only two small classrooms in one rural school, carriage results may not accurately reflect this age-group in the general population. While carriage could be unusually high on the day of saliva sampling, reflecting a micro-epidemic reported by Leino *et al*. [40], this seems an unlikely scenario as no particular serotype dominated in carriage, and neither was an increase in respiratory symptoms noticed before or after sample collection. Interestingly, Weinberger *et al*. [41] recently reported on an increase in carriage prevalence and IPD incidence in children below school age which coincided with older children returning to school after the summer break. It may support the presence of a significant reservoir of *S. pneumoniae* in asymptomatic schoolchildren that affects carriage and disease dynamics in the whole population.

The major findings of this study are the high rate of pneumococcal carriage in saliva, the high number of serotypes present and the high rate of multiserotype carriage in asymptomatic schoolchildren. It suggests a larger than contemporarily reported circulation of *S. pneumoniae* in this age group and weaker than expected competitive interaction between multiple strains colonising a single host. The simplicity of saliva collection and the high sensitivity of pneumococci detection suggest that saliva could be considered as a sampling method in future pneumococcal surveillance studies, either in combination with accepted standard methods, or as an alternative to.

## Supporting Information

Figure S1 **Effect of the culture-enrichment step on extracellular pneumococcal DNA.**
(PDF)

Figure S2 **Correlations between the number of serotypes detected, absolute abundance of** ***S. pneumoniae*** **and the total bacterial load.**
(PDF)

Table S1 **Serotyping results for all 50 schoolchildren.**
(PDF)
