# Supplementary material for: Streptococcus pneumoniae in Saliva of Dutch Primary School Children
Source: PLoS One. 2014 Jul 11;9(7):e102045. doi: 10.1371/journal.pone.0102045 (PMC4094488; doi:10.1371/journal.pone.0102045)
Supplement: Figure S1 — Effect of the culture-enrichment step on extracellular pneumococcal DNA. (PDF) [file pone.0102045.s001.pdf]

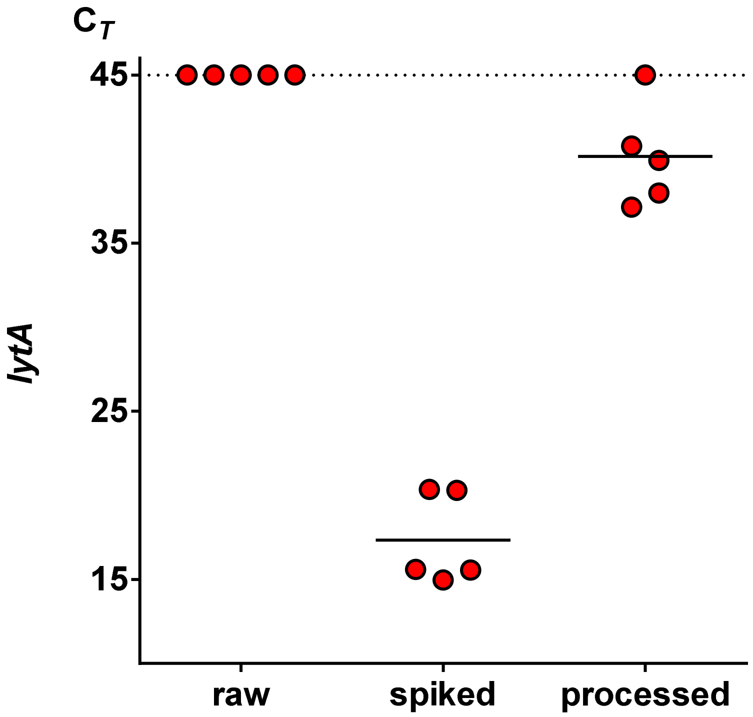

FIGURE S1. Effect of the culture-enrichment step on extracellular pneumococcal DNA.

Frozen saliva samples (200 µl) from five individuals identified as non-carriers were thawed on ice, vigorously vortexed for 30 seconds and half of the volume transferred to new tube. The remaining 100 µl volumes were spiked with 10 µl of DNA extracted from a S. *pneumoniae* strain, vortexed vigorously for 20 seconds and 10 µl cultured on SB7-Gent plate for culture-enrichment as described in the Materials and Methods. Remaining volumes of spiked samples were stored frozen along raw saliva samples.

Next, DNA was extracted from raw and spiked saliva and from culture-enriched (processed) samples and tested in *lytA*-specific qPCR as described in the Materials and Methods. Each dot represents the *lytA* C_T for individual samples tested. Horizontal lines represent median C_T per set of samples. Dotted line marks the total number of 45 cycles in qPCR reaction. Note the decline in *lytA* signal strength (reflected by an increase in C_T) observed in culture-enriched compared to saliva samples spiked with pneumococcal DNA.
