# Supplementary material for: Streptococcus pneumoniae in Saliva of Dutch Primary School Children
Source: PLoS One. 2014 Jul 11;9(7):e102045. doi: 10.1371/journal.pone.0102045 (PMC4094488; doi:10.1371/journal.pone.0102045)
Supplement: Figure S2 — Correlations between the number of serotypes detected, absolute abundance of S. pneumoniae and the total bacterial load. (PDF) [file pone.0102045.s002.pdf]

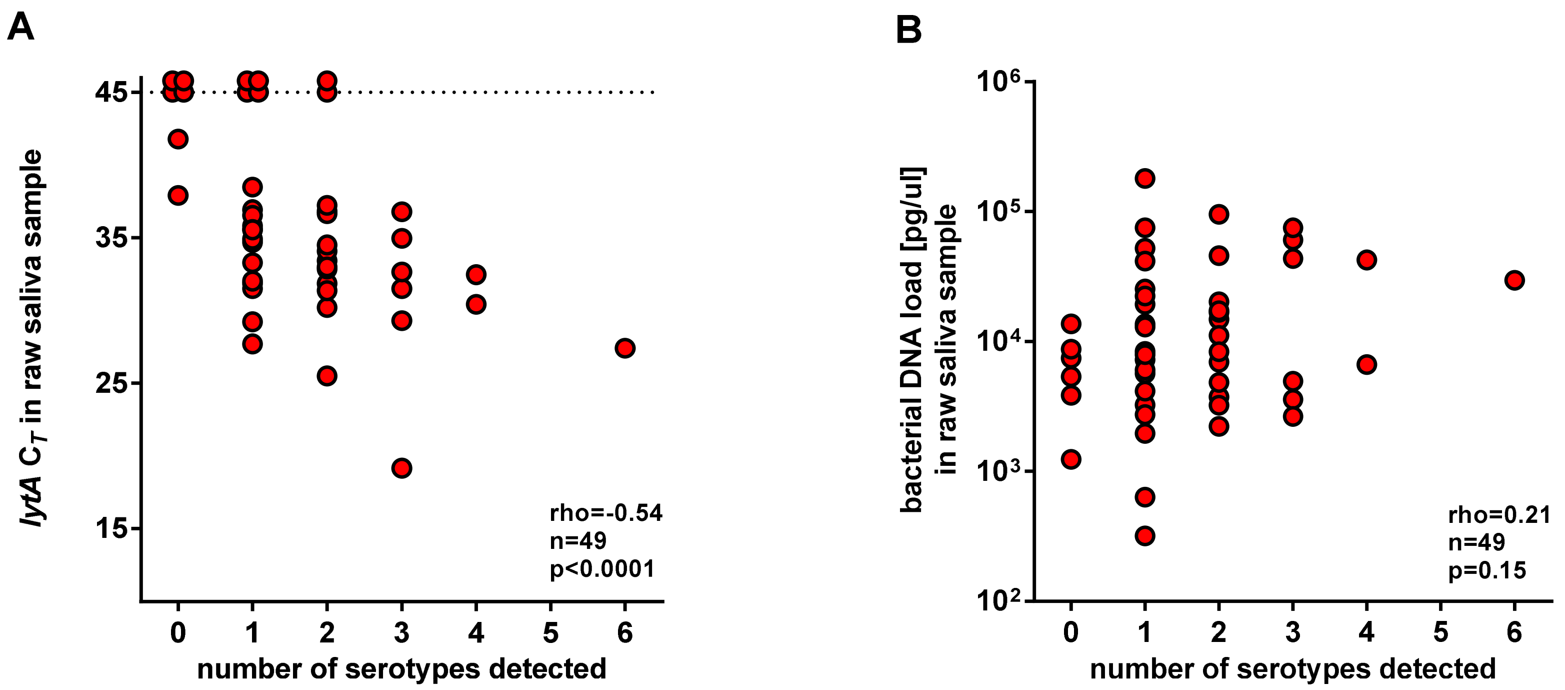

FIGURE S2. Correlations between the number of serotypes detected, absolute abundance of *S. pneumoniae* and the total bacterial load.
Spearman's rank correlations coefficients (rho) between the number of serotypes/strains detected per saliva sample in the study and **A.** quantity of *S. pneumoniae* determined in *lytA*-specific qPCR and **B.** bacterial DNA total load as determined using 16S based qPCR in DNA extracted from 49 uncultured (raw) samples (one sample was unavailable for 16S analysis). Each dot represents an individual sample. The negative rho in **A.** represents a positive correlation between parameters as the lower C_T, the higher the number of copies of the sequence targeted in the assay (the lower value of C_T, the stronger the signal detected).
Correlation coefficients were also calculated only for the subset of samples classified as positive for *S. pneumoniae*. The exclusion of samples negative for pneumococcus could be considered appropriate in order to account for the potential bias caused by samples from non-carriers. After exclusion of all six samples classified in the study as negative for *S. pneumoniae* (zero serotypes detected in a sample), the correlations between the overall number of serotypes/strains detected and strength of *S. pneumoniae* specific signal remained significant for both *lytA* (rho=-0.38, n=43, p=0.011) and *piaA* (rho=-0.44, n=43, p=0.003; not shown) whereas the correlation between the overall number of serotypes/strains detected and the sample total bacteria load remained not significant (rho=0.14, n=43, p=0.38).
