# Supplementary material for: Streptococcus pneumoniae in Saliva of Dutch Primary School Children
Source: PLoS One. 2014 Jul 11;9(7):e102045. doi: 10.1371/journal.pone.0102045 (PMC4094488; doi:10.1371/journal.pone.0102045)
Supplement: Table S1 — Serotyping results for all 50 schoolchildren. (PDF) [file pone.0102045.s003.pdf]

	1	2	3	4	5	6A/B	6C/D	7A/F	7C/B/40	8	9A/V	9N/L	10A(/B)	10/F/C/33C	11A/D	12F/A/B(/44/46)	13	14	15(A/F)(B/C)	16F	17F	18B/C(A/F)	19A	19F(/B)	20	21	22A/F	23A	23B	23F	24F/A/B	31	34	33A/F(37)	35A/C/42	35B	35F/47F	38/25F/A	39	NT
1																																		b						
2																				c																				
3															c	q							b				q													
4																																								
5							b																																	
6													c																											
7			b																b	c																				
8																																		b						
9																																		q						
10																																								
11																														b				b						
12																																								
13							q																																	
14																																								1
15																																								
16			q													q																								
17						b										q																								
18																																								
19																																								
20																		q					q																	
21																																								1
22															c	q																								
23																q		q					q																	

24									q								q																						
25																													c											
26																q			q																					
27			q																																					
28			q																b				q																	
29													b						b	c																	c			
30																		q	q																					
31							b				b																													
32																			b																					
33																q																		b						
34						b																																		
35																																								1
36						b																																		
37																				c			b																	
38	q															q																								
39																			q																					
40			b																																					
41							q								c				b																					
42			b																																					
43																																		q						
44			b																				b																	
45							q												b															b						
46																										c														
47							b	b											b	c			b											b						
48																																		q						
49			b				b																b																	
50			c			b																																		

TABLE S1. Serotyping results for all 50 schoolchildren are depicted.

Shown are the results considered positive in the molecular assays for sample serotype determination and included in the study analysis as determined by qPCR only (q), cPCR only (c) or when both molecular assays were in agreement (b). Letters in bold represent molecular serotyping results were confirmed by the Quellung method. Squares coloured green indicate samples from which the cPCR amplicon generated had full homology (no single nucleotide mismatch detected) with the published or control sequence for the serotype-specific assay. Squares coloured grey indicate samples from which the cPCR amplicon generated did not have full homology (one or more nucleotide mismatches) and were excluded from the study results. Individuals denoted in grey text were negative for *S. pneumoniae*. Serotypes coloured yellow indicate those tested for by the cPCR/hybrid method only.
